# Phonon Involved Photoluminescence of Mn^2+^ Ions Doped CsPbCl_3_ Micro‐Size Perovskite Assembled Crystals

**DOI:** 10.1002/advs.202413402

**Published:** 2025-01-22

**Authors:** Jialiang Gao, Yangyang Guo, Xiuhai Zhang, Lu Liu, Huixin Li, Zeyi Cheng, Peng Liu, Fan Dong, Jiandong Wu, Taihong Liu, Huaming Sun, Miao Zhang, Hervé Aubin, Hongyue Wang, Hongqiang Wang

**Affiliations:** ^1^ State Key Laboratory of Solidification Processing Center for Nano Energy Materials School of Materials Science and Engineering Northwestern Polytechnical University and Shaanxi Joint Laboratory of Graphene (NPU) Xi'an 710072 P. R. China; ^2^ Key Laboratory of Applied Surface and Colloid Chemistry Ministry of Education School of Chemistry and Chemical Engineering Shaanxi Normal University Xi'an 710119 P. R. China; ^3^ Materials Institute of Atomic and Molecular Science Shaanxi University of Science and Technology Xi'an 710021 P. R. China; ^4^ Department of Nanoelectronics Center for Nanoscience and Nanotechnology (C2N) CNRS University Paris‐Saclay 91120 France

**Keywords:** electron–phonon coupling, Mn^2+^ ions, perovskite nanocrystals, self‐assembly

## Abstract

Mn^2+^ ions doped CsPbCl_3_ perovskite nanocrystals (NCs) exhibit superiority of spin‐associated optical and electrical properties. However, precisely controlling the doping concentration, doping location, and the mono‐distribution of Mn^2+^ ions in the large‐micro‐size CsPbCl_3_ perovskite host is a formidable challenge. Here, the micro size CsPbCl_3_ perovskite crystals (MCs) are reported with uniform Mn^2+^ ions doping by self‐assembly of Mn^2+^ ions doped CsPbCl_3_ perovskite NCs. The electron–phonon coupling strength is enhanced in the perovskite self‐assembled CsPbCl_3_ MCs, which remarkably accelerates the PL decay of Mn^2+^ ions in room temperature. Furthermore, the phonon‐involved PL emission splits to two peaks at low temperature of 80 K, due to the phonon emission and absorption‐induced energy exchange for exciton recombination in Mn^2+^ ions. These findings not only demonstrate a novel material system but also introduce a new theoretical framework for phonon‐modulated PL manipulation in Mn^2+^‐doped perovskite materials.

## Graphical abstract

Self‐assembled MCs of Mn^2+^: CsPbCl_3_ are presented and investigated the influence of enhanced electron–phonon coupling on the carrier dynamics of the host perovskite and Mn^2+^ dopants in MCs, which exhibits accelerated PL lifetime at room temperature and phonon‐involved PL splitting at low temperature of 80 K.

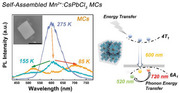

## 1 Introduction

Magnetic‐ion‐doped perovskite nanocrystals (NCs) have shown potential in application such as spintronic,^[^ ^1^, ^2^ ^]^ bioimaging,^[^ ^3^ ^]^ information storage,^[^ ^4^ ^]^ and anti‐counterfeiting,^[^ ^5^ ^]^ owing to their superior dopant‐associated optical and electrical properties.^[^ ^6^ ^]^ In particular, Mn^2+^ doped perovskite NCs show characteristic PL emission that comes from the electrical d‐d transition of Mn^2+^ ions in the ligand field of host lattice.^[^ ^7^, ^8^ ^]^ Since the d‐d transition of Mn^2+^ is a spin‐flip process, the direct exciting in Mn^2+^ ion is spin‐forbidden.^[^ ^9^, ^10^ ^]^ Thus, the excitation of Mn^2+^ dopants (from its states of ^6^A_1_ to ^4^T_1_) need energy transfer from excited host, then the carrier undergoes spin‐flip again to emit feature photon with energy difference between ^6^A_1_ and ^4^T_1_.^[^ ^11^, ^12^ ^]^ Since the recombination of the exciton in Mn^2+^ ions needs the spin‐flipping, which leads to long PL lifetime in milli‐second scale, as well as the phonon involved broadening PL linewidth.^[^ ^4^ ^]^ Thus, the electron–phonon coupling could affect the carrier kinetic and PL properties of Mn^2+^ dopants and perovskite host. It has been widely demonstrated that perovskites show strong electron–phonon coupling associated PL emission,^[^ ^13^ ^]^ due to the soft lattice of perovskites, which facilitates strong phonon vibrations. For instance, strong electron–phonon coupling in perovskite lattice induces the self‐trap state that is responsible of the broadening of PL spectrum covering the whole visible window.^[^ ^1^, ^14^ ^]^ Furthermore, the surface phonon modes dominate the PL linewidth of CsPbBr_3_ perovskite NCs, and the size of the perovskite NCs could strongly affect the coupling strength of electron and the surface phonon.^[^ ^15^ ^]^ Therefore, the strength of electron–phonon coupling is varied in the perovskite NCs in different dimensions, which also provides a possibility to control the PL emission of Mn^2+^ ions doped perovskite through changing the electron–phonon coupling strength in the perovskite NCs with different size.

Although this size‐dependent electron–phonon coupling is of significant interest for developing new method to modulate the carrier kinetic and PL emission of Mn^2+^ ions doped perovskite, the magnetic coupling state of Mn^2+^ ions and the distinct ligand field environment still remarkably affect its PL emission and are hardly distinguished.^[^ ^7^, ^10^ ^]^ For instance, by increasing the doping concentration, the interaction of two near Mn^2+^ ions are increased to form a magnetic coupling state between Mn^2+^ ions, showing accelerated PL lifetime or even quenched PL emission.^[^ ^2^, ^16^ ^]^ Moreover, the different locations of Mn^2+^ ions (on the surface or inside of the perovskite NCs) exhibit distinct PL decay due to the different ligand field environment.^[^ ^4^ ^]^ In the most of cases, the PL emission of Mn^2+^ ions doped perovskite NCs is manipulated by varying the doping concentration or the doping locations.^[^ ^17^, ^18^ ^]^ However, the competition of substitution between Pb^2+^ and Mn^2+^ ions are fierce during the fast crystallization processes of perovskite NCs, and the self‐cleaning of perovskite could squeeze the Mn^2+^ impurity out of the lattice.^[^ ^2^ ^]^ Therefore, to eliminate the interference from the magnetic coupling state of Mn^2+^ ions and the distinct ligand field environment, studies on the electron–phonon manipulated PL emission of Mn^2+^‐doped perovskite NCs need to ensure the exact distribution of Mn^2+^ ions in perovskite NCs of different sizes, particularly across the nano‐to‐micro scale, which remains a formidable challenge.

Here, we show that the micro‐size CsPbCl_3_ perovskite crystals (MCs) obtained by self‐assembly of Mn^2+^ ions doped CsPbCl_3_ perovskite NCs. The MCs exhibit homogeneous distribution of Mn^2+^ dopants same as the CsPbCl_3_ perovskite NCs, showing identical PL emission and magnetic coupling state of Mn^2+^ ions with the CsPbCl_3_ perovskite NCs at room temperature. We found that the strength of electron–phonon coupling is enhanced in this large size of self‐assembled CsPbCl_3_ MCs, which remarkably changes the carrier kinetic of Mn^2+^ ions for PL emission, showing accelerated PL decay at room temperature and phonon‐involved PL splitting at low temperature of 80 K. These two split PL peaks come from the energy exchange between the free exciton and phonon (phonon absorption and phonon emission) during exciton recombination. This phonon‐modulated carrier kinetic pioneers a novel channel to manipulate the PL emission of Mn^2+^ doped perovskite materials.

## 2 Results and Discussion

**Figure 1:**
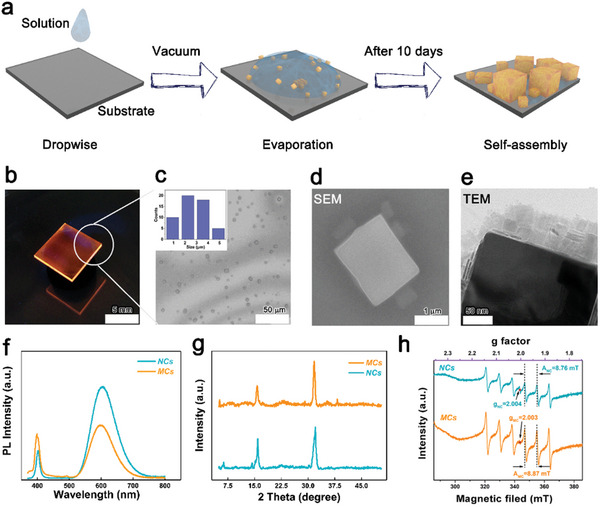
a) Scheme of the self‐assembly processes. b–e) The optical image, SEM, and TEM images of self‐assembled nanocrystals. Inset: The statistical size distribution of the MCs. f–h) The PL, XRD, and EPR spectra of perovskite NCs and self‐assembled MCs.

The Mn^2+^ ions doped CsPbCl_3_ perovskite NCs were synthesized through ultrasonication‐assisted method,^[^ ^19^, ^20^ ^]^ See the details in the Experimental Section. Figure S1 (Supporting Information) shows the XPS spectra of CsPbCl_3_ NCs with and without Mn^2^⁺ doping. Figure S1a (Supporting Information) presents the spectrum for Mn^2+^‐doped CsPbCl_3_ NCs, where a peak at ≈645 eV corresponds to the Mn 2p core levels, confirming Mn^2^⁺ incorporation. Figure S1b,c (Supporting Information) shows spectra for Pb and Cl, where the peaks for both elements shift slightly in the doped sample compared to the undoped reference. These shifts suggest changes in the local chemical environment due to Mn^2+^ incorporation and are attributed to the substitution of Pb^2+^ by Mn^2+^ in the crystal lattice. A corresponding schematic illustration of the Mn^2+^‐doped CsPbCl_3_ is provided in Figure S2 (Supporting Information). Figure S3 (Supporting Information) shows the PL, absorption spectra, and TEM images of the NCs, the characteristic PL emission of Mn^2+^ ions with (photoluminescence quantum yield) PLQY of ≈58.5% can be observed, and the feature band edge of CsPbCl_3_ is also shown on the absorption spectrum at wavelength of ≈400 nm.^[^ ^21^, ^22^ ^]^ The mono‐dispersed perovskite NCs show uniform cubic shape with mean size of ≈10 nm, this mono‐dispersion of perovskite NCs enables the success of followed self‐assembly.^[^ ^23^, ^24^ ^]^ The NCs contained toluene solution was dropped on a glass substrate, which placed in a petri dish, then immediately encapsulate the petri dish with a plastic bag following with pumping to achieve a vacuum of −0.1 MPa for 120 s, placing the encapsulated petri dish in an oven with a constant temperature of 25 °C to achieve the self‐assembled MCs. We carried out the self‐assembly processes at room temperature of 25 °C to sustain the same Mn^2+^ ion distribution in the perovskite NCs properly. Because the speed of self‐assembly process is not linear to the time batch by batch, and the self‐assembly process will be terminated once opening the sample, we thus keep the samples in the oven for over 10 days to make sure the success of self‐assembly. (see the details in the Experimental Section). The scheme of the self‐assembly process is shown in **Figure** **1**a. Under such condition of low vacuum, a small amount of solvent left on the substrate, forming many small and separated droplets on the glass. In these small droplets, the NCs aggregated and formed perovskite self‐assembly gradually under the driven force of increased concentration during the process in oven for over 10 days (Figure S4, Supporting Information).

Figure 1b–e shows the optical, SEM, and TEM images of the self‐assembly samples. The sample shows characteristic PL emission of Mn^2+^ ion with orange color (Figure 1b). The assembled MCs are dispersed on glass substrate homogeneously (Figure 1c). The inset in Figure 1c shows that the statistical size distribution of the MCs ranges from 1 to 5 µm. The SEM image of MCs in Figure 1d shows a MCs with cubic size of ≈1.5 µm. From TEM image in Figure 1e, many assembled nanoplates are found in the vicinity of the assembled MCs, owing to the solvent treatment during the TEM sample preparation. The MCs cannot be formed on the copper grid directly, we just wash the self‐assembly sample with anti‐solvent of toluene or hexene to transfer the MCs from the glass substrate into solution, then followed with typical TEM sample preparation. The perovskite NCs could regrowth or recrystallization during this solvent treatment to generate such nanoplates.^[^ ^28^ ^]^ To avoid the influence of the regrowth nanoplates, the followed optical characterization is conducted on the MCs samples on glass without any solvent treatments.

Moreover, we measured the Mn^2+^ concentration of NCs and MCs, as shown in Table S1 (Supporting Information), the ratio of Mn^2+^/ Pb^2+^ in the NCs and MCs are similar. We highlight here that the superlattice of self‐assembled NCs were supposed to be formed in this process based on the references,^[^ ^23^, ^25^ ^]^ however, we have never observed such superlattice structure from our samples. Thus, in this work, we would like to rigorously define the self‐assembly of the NCs as MCs rather than superlattice.^[^ ^26^, ^27^ ^]^ From Figure 1f–h and Figure S5 (Supporting Information), the PL, XRD, EPR and even PLE data of the NCs are not significantly shifted after assembling to MCs for 10 days, only showing a decreased PLQY (≈39%) in self‐assembled MCs (Figure S6, Supporting Information) because of the unavoidably increased defects of the perovskite that reduces the energy transfer efficiency between perovskite host and Mn^2+^ dopants. Therefore, we propose that the self‐assembly processes from NCs to MCs will not change the component of host perovskite, and the distribution of Mn^2+^ dopants in such large size of MCs is sustained same as the perovskite NCs, which is facilitated to study the electron–phonon coupling involved PL emission of the Mn^2+^ doped MCs. We need to highlight that the dual PL emission is not related to the size inhomogeneity, the size distribution of the MCs is a single Gaussian distribution in the inset of Figure 1c, if the dual PL emission is caused by the size inhomogeneity, the corresponding dual Gaussian distribution for size rather than a single distribution is needed. The linewidth of XRD signal of self‐assembled MCs at 2θ of ≈32 ° is only slightly narrow by comparing with that of perovskite NCs, indicating the slight increased grain size of MCs. To sustain the same Mn^2+^ distribution in MCs, we conducted the self‐assembly processes in room temperature, only local crystallization could be occurred partially in the MCs during the 10 days of self‐assembly,^[^ ^29^ ^]^ which might be the reason that we did not observe the superlattice structure with TEM characterization and the linewidth of XRD signal is only slightly narrow. Notably, we extracted the g factors and hyperfine coupling constants of NCs and MCs, as shown in Figure 1h. NCs exhibit g factor of 2.004 with hyperfine coupling constant of A_NC_ = 8.76 mT, and MCs have g factor of 2.003 with hyperfine coupling constant of A_MC_ = 8.87 mT, respectively. Both g factors of NCs and MCs are closed to 2 and these EPR spectra show typical six hyperfine splitting, indicating the Mn^2+^ located in the octahedral environment in perovskite lattice with small g‐anisotropy, which is consistent with literatures.^[^ ^30^, ^31^ ^]^ MCs have slightly larger hyperfine coupling constant, which is probably due to the uncoordinated Mn^2+^ on the surface of NCs transferring into the inner of perovskite lattice of MCs after self‐assembly, the fully coordinating environment increases the ionicity of Mn^2+^ to enlarge the hyperfine coupling constant.^[^ ^32^ ^]^

We also conducted the temperature‐dependent EPR measurements, as shown in Figure S7 (Supporting Information). NCs and MCs were measured in hexene solution. NCs show typical evolution of EPR spectra, only the EPR intensity are decreased with cooling down the temperature, the g factor and hyperfine coupling constant are not changed significantly. However, MCs show ruleless evolution of EPR spectra after the temperature decreasing to 270 K, probably because the self‐assembled MCs are disassembled or aggregated in the solution.

**Figure 2:**
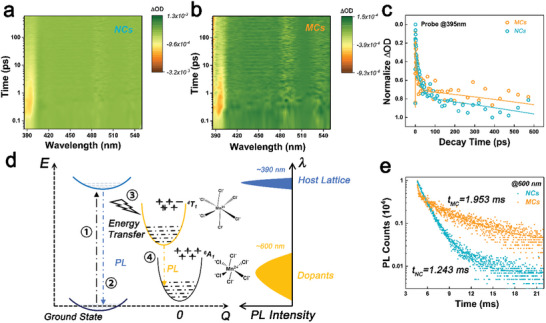
a,b) TA spectra of perovskite NCs and MCs (probe 380–550 nm). c) TA decay and fitting curves of perovskite NCs and MCs. d) Diagram of excitation, energy transfer, and recombination in Mn^2+^ doped CsPbCl_3_. e) TRPL data and fitting curves of Mn^2+^ ions doped perovskite NCs and MCs.

To study the electron–phonon coupling involved energy transfer kinetic between host perovskite lattice and Mn^2+^ ions, we conducted femtosecond transient absorption (TA) experiments. Since the direct excitation of Mn^2+^ ion is spin‐forbidden, only host perovskite signal can be detected at wavelength of ≈390 nm, as shown in **Figure** **2**a,b. We did not observe the significant difference on the TA data of NCs and self‐assembled MCs, as well as the decay kinetic spectra (Figure 2c; Table S2, Supporting Information). Therefore, the NCs and self‐assembled MCs exhibit almost same carrier kinetic in the host perovskite, the carrier kinetic includes the processes of excitation, recombination in perovskite host and energy transfer from perovskite host to Mn^2+^ dopants, which are corresponded to schematic processes of ①, ②, and ③ in Figure 2d. However, the radiative recombination decay of Mn^2+^ dopants (process of ④ in Figure 2d) shows remarkably difference between the self‐assembled MCs and NCs, the time‐resolved PL (TRPL) data of NCs and self‐assembled MCs are shown in Figure 2e. By comparing with NCs, self‐assembled MCs show significantly different PL decay that apparently consists of two components of radiative recombination in Mn^2+^ ions, one is faster and the other one is slower than that of NCs.

Since the exciton recombination in Mn^2+^ ions is spin‐forbidden, the PL of Mn^2+^ ions typically exhibits mono‐exponential decay with lifetime as long as in millisecond when the isolated state Mn^2+^ ions are located in the fully coordinated ligand field of the host lattice.^[^ ^10^ ^]^ For the as‐prepared NCs, some Mn^2+^ ions are located on the surface of NCs, those Mn^2+^ ions are partially coordinated with the perovskite host lattice. Then, in the self‐assembled MCs, the NCs are melted and the interfaces are disappeared, which lead to those partially coordinated Mn^2+^ ions becoming fully coordinated inside of the perovskite lattice. Since the specific surface of self‐assemble MCs is exponentially decreased by comparing that of perovskite NCs, the concentration of Mn^2+^ ions on the surface of NCs is decreased significantly, and transferring into a completed coordination environment, leading to the ratio of long lifetime component raises in the PL decay by comparing to the perovskite NCs (Figure 2e). Furthermore, the exciton lifetime of Mn^2+^ ion is independent with the host defect, but more sensitive to the magnetic coupling state of Mn^2+^ ions. As shown in Figure 2e and Table S3 (Supporting Information), the self‐assembled MCs also show a fast radiative decay component (0.248 ms) with a heavy weight over 50% by comparing to perovskite NCs. Since we have demonstrated above that the host perovskite lattice and the distribution of Mn^2+^ ions are not changed between NCs and self‐assembled MCs, this fast PL decay should not originate from the recombination channel of magnetic coupled Mn^2+^ ions. Super‐fluorescence is a characteristic emission with fast radiative decay in self‐assembled superlattice, which exhibits coherent photon emission at helium temperature (≈4 K), showing red‐shifted emission with narrow linewidth.^[^ ^23^, ^33^, ^34^ ^]^ Thus, it is also impossible to excite super‐fluorescence in the self‐assembled MCs in room temperature in this work. Then, excluding the magnetic coupling state of Mn^2+^ ions and the super‐fluorescence,^[^ ^35^, ^36^ ^]^ we propose that the fast decay component of TRPL in Figure 2e might come from the enhanced electron–phonon coupling in the self‐assembled MCs, which also induces the accelerated recombination due to the superposed wavefunction between exciton and phonons in the lattice.^[^ ^35^, ^36^ ^]^

**Figure 3:**
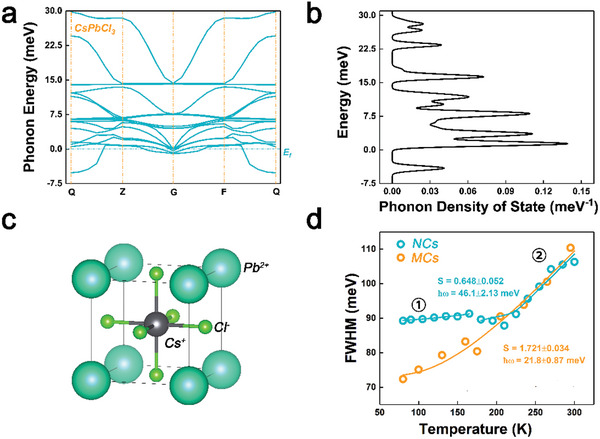
a) CsPbCl_3_ phonon band structure. b) CsPbCl_3_ phonon density of state. c) The optimized geometry unit for DFT calculation. d) The Boson model fitting curves of host perovskite lattice.

We need to highlight here that the PL emission of Mn^2+^ ions including peak wavelength, full width at half maximum (FWHM), and intensity are remarkably sensitive to temperature, which hampers the extraction of electron–phonon coupling information of the Mn^2+^ ions from the temperature‐dependent PL spectra. We thus first studied electron–phonon coupling of the exciton of host perovskite in NCs and self‐assembled MCs. We calculated the phonon dispersion and phonon density of state of host CsPbCl_3_ perovskite through density functional theory (DFT), as shown in **Figure** **3**a,b, and the calculation was conducted based on the geometry optimization from CsPbCl_3_ perovskite unit, as shown in Figure 3c. The phonon energy of the perovskite lattice is in the range of 0–30 meV, which is consistent with references.^[^ ^36^, ^37^ ^]^ Then, we conducted temperature‐dependent PL measurements of the perovskite NCs and MCs, the PL evolution of host CsPbCl_3_ perovskite is shown in Figure S8 (Supporting Information). Then we extracted the PL FWHM data of the host perovskite from Figure S8 (Supporting Information), and fitted with Boson model:^[^ ^14^ ^]^

(1) $$\Gamma \left(T\right)=\Gamma_{0}+\sigma T+\Gamma_{LO}\frac{1}{e^{E_{LO}/k_{B}T}-1}$$

where Γ_0_represents the phonon emission linewidth of the material at 0K, which is associated with the host lattice scattering properties. σdenotes the exciton‐acoustic phonon coupling coefficient. Γ_*LO*_ is exciton‐longitudinal optical phonon coefficient that dominates the linewidth broadening at high temperature. *E_LO_* is the phonon energy, *k_B_* represents the Boltzmann constant. *T* is temperature. The fitting results are shown in Figure 3d and **Table** **1**. The fitting of PL FWHM data of perovskite NCs contains two regimes of (①: 80–180 K) and (②: 180–300 K). For fitting at low‐temperature regime ①, it yields electron–phonon coupling coefficient of Γ_*LO*1_ ≈18.6 meV with phonon energy of *E*_*LO*1_ ≈9.9 meV, while for fitting at high temperature regime ②, it yields electron–phonon coupling coefficient of Γ_*LO*2 _≈82.6 meV with phonon energy of *E*_*LO*2_ ≈41.0 meV. In the perovskite NCs, by decreasing temperature, the electron–phonon coupling becomes weak and trends to have coupling to phonons with low energy, which is consistent to the theoretical result from reference,^[^ ^15^ ^]^ where the size of NCs could significantly affects the electron–phonon coupling. In this work, the size of perovskite MCs is much larger than the size of NCs, the corresponded electron–phonon coupling should be stronger in the MC. For the fitting data of MCs, the fitting yields electron–phonon coupling coefficient of Γ_*LO*3_≈141.8 meV with phonon energy of *E*_*LO*3_ ≈16.3 meV. The perovskite MCs exhibits much larger electron–phonon coupling coefficient than that of NCs, even though the evolution of PL FWHM of NCs and MCs are superposed at high‐temperature regime (180–300 K). The electron–phonon coupling becomes stronger in the MCs, which might be due to the increased defect density in the MCs or the coherent resonance of the phonons in the MCs.

**Table 1:** Boson model fitting data of Mn2+ ions doped perovskite NCs and MCs.

	*NCs*		*MCs*
	①	②	/
Γ_0_(meV)	*93.8*	*73.5*	*73.4*
Γ_*LO*_(meV)	*18.6*	*82.6*	*141. 8*
*E_LO_* (meV)	*9.9*	*41.0*	*16.3*

We also extracted Huang‐Rhys factor (S) from the PL FWHM data (Figure S9, Supporting Information). For perovskite NCs, the Huang‐Rhys factor of S1 is of ≈0.65, and for self‐assembled MCs, the Huang‐Rhys factor of S2 is of ≈1.72, indicating NCs is in the weak electron–phonon coupling regime and self‐assembled MCs is in the strong electron–phonon coupling regime,^[^ ^34^ ^]^ which are consistent with the fitting results from Boson model. Therefore, the observed fast PL decay component in self‐assembled MCs in Figure 2e originates from the enhanced electron–phonon coupling.

**Figure 4:**
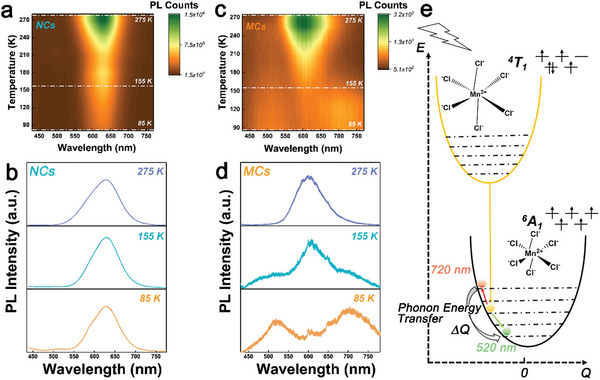
a,b) Temperature‐dependent PL of Mn^2+^ ions in perovskite NCs. c,d) Temperature‐dependent PL of Mn^2+^ ions in perovskite MCs. Three PL spectra in Figure 4b,d are extracted from Figure 4a,c (indicated by white dash lines) at three measurement temperatures of 275, 155, and 85 K, respectively. e) Scheme of the phonon involved splitting PL emission in Mn^2+^ ions doped perovskite MCs.

We further found that the enhanced electron–phonon coupling not only accelerates the recombination rate of exciton of Mn^2+^ dopants, but also remarkably affects its PL emission peak at low temperature. By decreasing the temperature from 300 to 80 K, the PL intensity of Mn^2+^ ion in perovskite NCs is gradually decreased, due to the energy transfer from perovskite host to Mn^2+^ dopant is hindered,^[^ ^38^ ^]^ as shown in **Figure** **4**,b. However, For the self‐assembled MCs, the intensity of PL emission at wavelength of ≈600 nm (2.07 eV) is also gradually decreased when the temperature is decreasing from 300 to 180 K, then, two peaks appear at wavelength of ≈520 nm (2.38 eV) and 720 nm (1.72 eV) when the temperature is cooling down below than ≈180 K, as shown in Figure 4c,d. Although CsPbCl_3_ NCs has phase transition at 193 K, this phase transition will not affect the emission of Mn^2+^ ion significantly, because the band gap of CsPbCl_3_ NCs only changed slightly from 1.969 to 1.973 eV,^[^ ^40^ ^]^ and the PL emission band of Mn^2+^ ion only depends on its own energy difference of ^6^A_1_ to ^4^T_1_ rather than the band gap of host lattice An analogous PL emission splitting is also observed in a different batch of perovskite MCs, as shown in Figure S10 (Supporting Information). Since the defect emission will be suppressed in low temperature due to the activity of electron transition from band to defect is prevented, the defect emission intensity should be decreasing with cooling down temperature.^[^ ^41^ ^]^ The two splitting PL emission (≈518 and ≈703 nm) intensity are increased with decreasing the temperature, the defect emission could be excluded. In the perovskite host lattice, Mn^2+^ ions located at the center of octahedral ligand field of Cl. It has been reported that the ligand nuclear distortion occurs in the excited Mn^2+^ ions coordinated ligand field,^[^ ^8^, ^39^ ^]^ leading to a coordinate shifting on the potential energy minimum between excited state of ^4^T_1_ and ground state of ^6^A_1_, as shown in Figure 4e. Because the excited state of ^4^T_1_ is sensitive to vibronic coupling of perovskite host ligand field, the PL emission from electron transition between ^4^T_1_ and ^6^A_1_ is remarkably broadening.^[^ ^1^, ^42^ ^]^ Moreover, Perovskite has been widely demonstrated as a characteristic material with feature of strong electron–phonon coupling, and this electron–phonon coupling can even be enhanced in the self‐assembled MCs as we discussed above. Therefore, the phonon energy involved PL emission of Mn^2+^ ions could be observed in the self‐assembled MCs. The phonon‐coupled PL emission, including both phonon energy emission and absorption‐associated electron transitions (green and red arrows, respectively), are depicted in Figure 4e. We found that the two PL peaks (520 and 720 nm) exhibit roughly equal energy difference of ≈0.33 eV relative to free exciton emission of 600 nm, indicating the approximal equal energy exchanging occurs to split the PL emission peak toward high energy and low energy sides simultaneously. Since these two PL peaks are broadening and the electron–phonon coupling is enhanced in the perovskite MCs, we propose that the two PL peaks are originated from strong phonons involved Stokes shift and anti‐Stokes shift, respectively, as shown in Figure 4e. Although the typical phonon modes of Mn^2+^ center and the host perovskite lattice are lower than 330 meV, the PL emission could be coupled with many phonons in 80 K in the electron–phonon coupling enhanced MCs, showing extremely broadening linewidth, as well as the large Stokes shift and anti‐Stokes shift simultaneously. We also suggest that the further studies should cool down the temperature to lower 4 K, the precise observation of Frank‐Condon effect could give more information referring the phonon involved PL emission from the perovskite MCs.

## 3 Conclusion

In summary, we presented a method for obtaining large size perovskite crystals with uniform distribution of Mn^2+^ dopants, by manipulating the mono‐dispersed perovskite NCs to form self‐assembled MCs in micro‐size. The self‐assembled MCs show same uniform distribution of Mn^2+^ ions in the perovskite host lattice by comparing with the perovskite NCs at room temperature. The results of theoretical calculation and temperature‐dependent PL reveal that the MCs exhibits enhanced electron–phonon coupling. We first demonstrated that the enhanced electron–phonon coupling in self‐assembled MCs remarkably affects the exciton recombination of Mn^2+^ dopants, showing accelerated PL lifetime in room temperature, as well as the phonon energy involved PL splitting at low temperature of 80 K. This work provides a new approach for manipulating the PL emission of Mn^2+^‐doped perovskite NCs and presents a novel finding regarding the phonon‐coupled carrier transitions in perovskite NCs

## Conflict of Interest

The authors declare no conflict of interest.

## Supporting information



Supporting Information

## Data Availability

The data that support the findings of this study are available from the corresponding author upon reasonable request.
