# Supplementary material for: Phonon Involved Photoluminescence of Mn2+ Ions Doped CsPbCl3 Micro‐Size Perovskite Assembled Crystals
Source: Adv Sci (Weinh). 2025 Jan 22;12(11):2413402. doi: 10.1002/advs.202413402 (PMC11923882; doi:10.1002/advs.202413402)
Supplement: Supplementary file 1 — Supporting Information [file ADVS-12-2413402-s001.docx]

***Supporting Information***

**Phonon Involved Photoluminescence of Mn^2+^ Ions Doped CsPbCl_3_ Micro-Size Perovskite Assembled Crystals**

Jialiang Gao^1,#^, Yangyang Guo^1#^, Xiuhai Zhang^1,#^, Lu Liu^2^, Huixin Li^1^, Zeyi Cheng^1^, Peng Liu^1^, Fan Dong^1^, Jiandong Wu^1^, Taihong Liu^2^, Huaming Sun^2^, Miao Zhang^3,^*, Hervé Aubin^4^, Hongyue Wang^1,^*, Hongqiang Wang^1,^*

^1^ State Key Laboratory of Solidification Processing, Center for Nano Energy Materials, School of Materials Science and Engineering, Northwestern Polytechnical University and Shaanxi Joint Laboratory of Graphene (NPU), Xi’an 710072, P. R. China.

^2^ Key Laboratory of Applied Surface and Colloid Chemistry, Ministry of Education, School of Chemistry and Chemical Engineering, Shaanxi Normal University, Xi'an, 710119, P. R. China.

^3^ Materials Institute of Atomic and Molecular Science, Shaanxi University of Science and Technology, Xi’an, 710021, P. R. China.

^4^Department of Nanoelectronics Center for Nanoscience and Nanotechnology (C2N), CNRS, University Paris-Saclay, France.

*E-mails: zhangmiao@sust.edu.cn, hongyue.wang@nwpu.edu.cn, hongqiang.wang@nwpu.edu.cn

^#^These authors are contributed equally

Keywords: Mn^2+^ ions, Perovskite Nanocrystals, Self-assembly, Electron-phonon coupling

**Experimental Section**

**Chemicals:** Manganese chloride tetrahydrate (MnCl_2_·4H_2_O), PbCl_2_ (Lead(II) chloride, 90%),1-octadence (ODE, 90%), oleylamine (OAm, 70%), oleic acid (OA, 90%), hydrochloric acid (HCl), Cesium carbonate (Cs_2_CO_3_, 99%). All chemicals were directly used without further purification.

**Synthesis:**

**Preparation of Cs-oleate (CsOA):**

Similarity to the previous method reported^1, 2^, cesium carbonate (Cs_2_CO_3_, 1.2 mmol), oleic acid (OA, 1.5 mL) and 1-octadence (ODE, 13 mL) were charged into a 25 mL three-neck flask. The mixture was degassed for 20 min in 90^o^C before heated in 150^o^C at vacuum. After cesium carbonate was completely dissolved, the solution was allowed to cool down to room temperature for further using.

**Preparation of oleylammonium chlorine (OAmCl):**

Hydrochloric acid (HCl, 1mL) and oleylamine (OAm, 10 mL) were combined a 25 mL three-neck flask with vigorously stirred and heated to 120^o^C under N_2_ flow. Then cooled to room temperature for further use.

**Preparation of Mn^2+^ doped CsPbCl_3_ perovskite nanocrystals:**

For a typical colloidal synthesis^3^, oleic acid (OA, 0.7 mL), oleylamine (OAm, 0.7 mL), oleylammonium chlorine (OAmCl, 0.5 mL, heated to 80^o^C) and 1-octadecene (ODE, 10 mL) were added to a constant amount of PbCl_2_-MnCl_2_ precursor powder and then processed by tip-sonication at a power of 240 W. During the sonication process, the colorless reaction medium gradually transformed from brown cloudy solution to pale yellow clear solution^.^ With the secondary injection of OA (0.7 mL) and OAm (0.7 mL), the solution became translucent, which marked the formation of L_2_[Pb_x_Mn_1-x_]Cl_4_. After 10 s, 0.5 mL Cs-oleate was swiftly injected into the reaction vessel, which resulted in the formation of Mn^2+^:CsPbCl_3_ NCs in <1 s. After the reaction was complete, the reaction medium was cooled down with ice-cold water and the unreacted precursors and excess ligands were removed by centrifugation at a speed of 10000 rpm for 10 min and then the particles were re-dispersed in 4 ml of toluene. After centrifuging the intermediate product at 2000 rpm for 10 min, the precipitate was dispersed in toluene for further self-assembly and characterization.

**Self-assembly of Mn^2+^ doped CsPbCl_3_ perovskite nanocrystals:**

For the growth of self-assembly doped nanocrystals, vacuum drying is used in combination with references^4, 5^. The experimental process for self-assembly involves dispensing 0.15 ml of nanocrystals toluene solution, which has been filtered through a PTFE filter, onto a 1.5 cm x 1.5 cm clean glass substrate (Note: the glass is cleaned by rinsing with piranha solution). The glass substrate is then placed into an aluminum foil-coated petri dish and subjected to vacuum drying to ensure a vacuum environment^6^. After approximately 10 days, the doped perovskite nanocrystals undergo self-assembly on the glass surface. All self-assembly preparations are conducted under vacuum or in an ambient environment.

**Characterization:**

UV−vis absorption spectra were measured on the Perkin-Elmer Lambda 35 UV-vis-NIR spectrophotometer. Photoluminescence and photoluminescence excitation spectra were recorded on an Edinburgh Instruments FLS980 fluorescence spectrometer (excitation at 350 nm). The SEM images of the nanocrystals were acquired using a field emission scanning electron microscope (FEI Nova) in secondary electron mode. The TEM images of nanocrystals were obtained by FEI Tecnai F30 microscope. XRD patterns were collected on Bruker D8 ADVANCE at 40 kV/30 mA using Cu Kαradiation (λ = 1.5418 Å). X-ray photoelectron spectroscopy (XPS) was conducted on a PHI Versa Probe II XPS system. The TRPL measurement were carried out with PicoQuant Fluo Time 300 with a semiconductor laser (excitation at 365 nm, catch at 600 nm). Photoluminescence quantum yield (PLQY) measurements were performed with the C11347 quantum yield measurement system from Hamamatsu Photonics Trading (China) Co., Ltd. The EPR/ESR measurements were carried out with Bruker E500 at 9.426 GHz (X band), using a field modulation frequency of 100 kHz and amplitude of 1 G. The EPR spectrometer was equipped with a temperature controller and liquid N_2_ cryostat for low-temperature measurements. Inductively coupled plasma optical emission spectroscopy (ICP-OES) analysis was carried out using a PerkinElmer 8300 ICP-OES instrument. Transient absorption spectroscopy results obtained on Helios-Ultrafast Systems (excitation at 350 nm, detection at 370-500 nm, 760 μW). Temperature dependent-PL was measured using OmniFluo900.

**Computational Method and Details**

The corresponding calculations were performed by using CASTEP, which is based on density functional theory. After the geometry optimization for CsPbCl_3_ with a symmetry group of $\text{Pm}\bar{3}\text{m}$ was conducted, the phonon dispersion and density of states were obtained by the finite displacement method. For electronic exchange-correlation interactions, the generalized gradient approximation (GGA) in the form of the Perdew-Burke-Ernzerhof (PBE) functional method was used. The energy cutoff was set as 285.7 eV, and the Monkhorst-Pack k-point grid was set as 4×4×4. During the geometry optimization process, 1×10^-5^ eV/atom, 0.03 eV/Å, 0.05 GPa, and 0.001 Å were set for energy, force, stress, and displacement convergence, respectively.

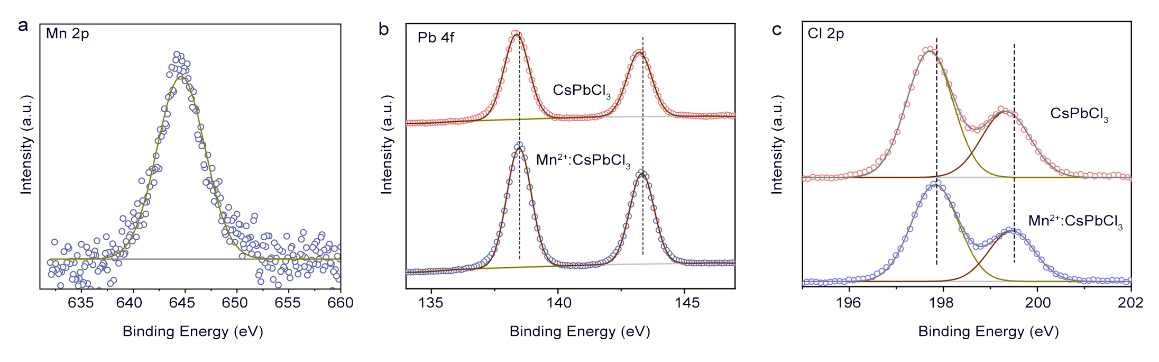


**Figure S1.** (a) XPS spectra for Mn 2p for Mn^2+^-doped CsPbCl_3_ NCs. XPS spectra for (b) Pb 4f and (c) Cl 2p for pristine and Mn^2+^-doped CsPbCl_3_ NCs.


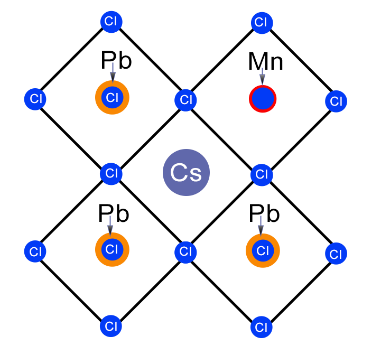


**Figure S2.** Schematic illustration of Mn^2+^-doped CsPbCl_3_.

**Table S1.** Characterization results of element content by ICP-OES.

| Samples | Mn^2+^ content (%) | Pb^2+^ content (%) | Mn^2+^/ Pb^2+^ (%) |
| --- | --- | --- | --- |
| NCs | 0.209 | 8.89 | 2.351 |
| MCs | 0.735 | 47.54 | 1.546 |


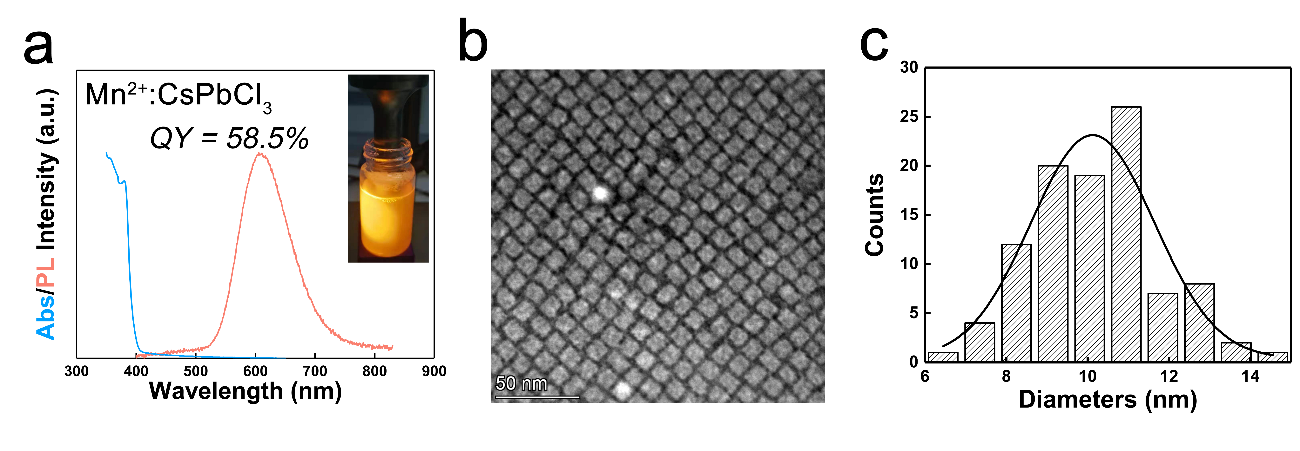


**Figure S3.** The (a) PL, absorption spectra, (b) TEM image and (c) statistical distribution of particle size of Mn^2+^: CsPbCl_3_ perovskite nanocrystals.


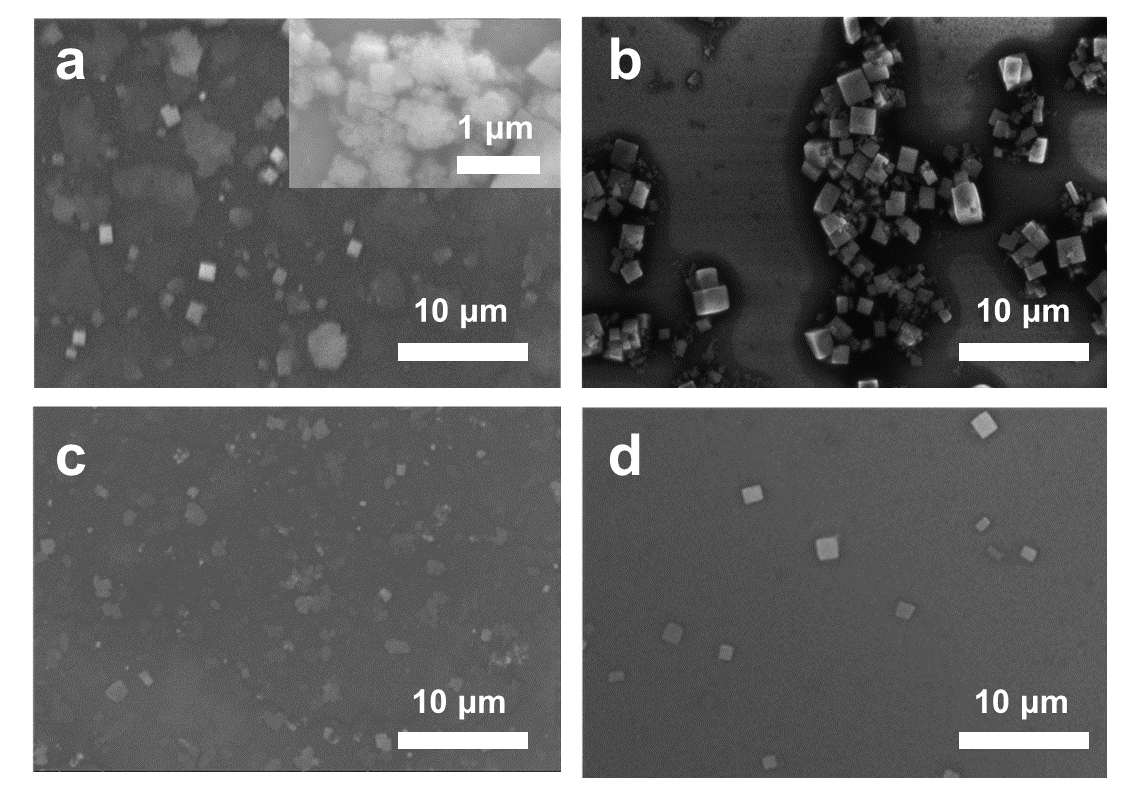


**Figure S4.** The SEM images of self-assembly samples at different timepoint. (a) Self-assembly process at 3 days from toluene. (b) Self-assembly process at 10 days from toluene. (c) Self-assembly process at 3 days from mixed solvent of toluene/hexene (1:1). (d) Self-assembly process at 10 days from mixed solvent of toluene/hexene (1:1).





**Figure S5.** PLE spectra of the Mn^2+^ doped NCs and the self-assembled MCs.


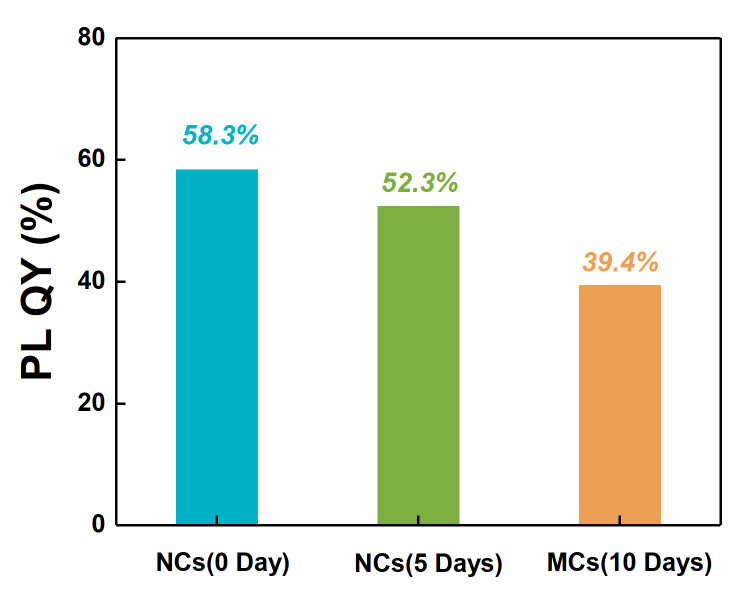


**Figure S6.** The PLQY of perovskite crystals.


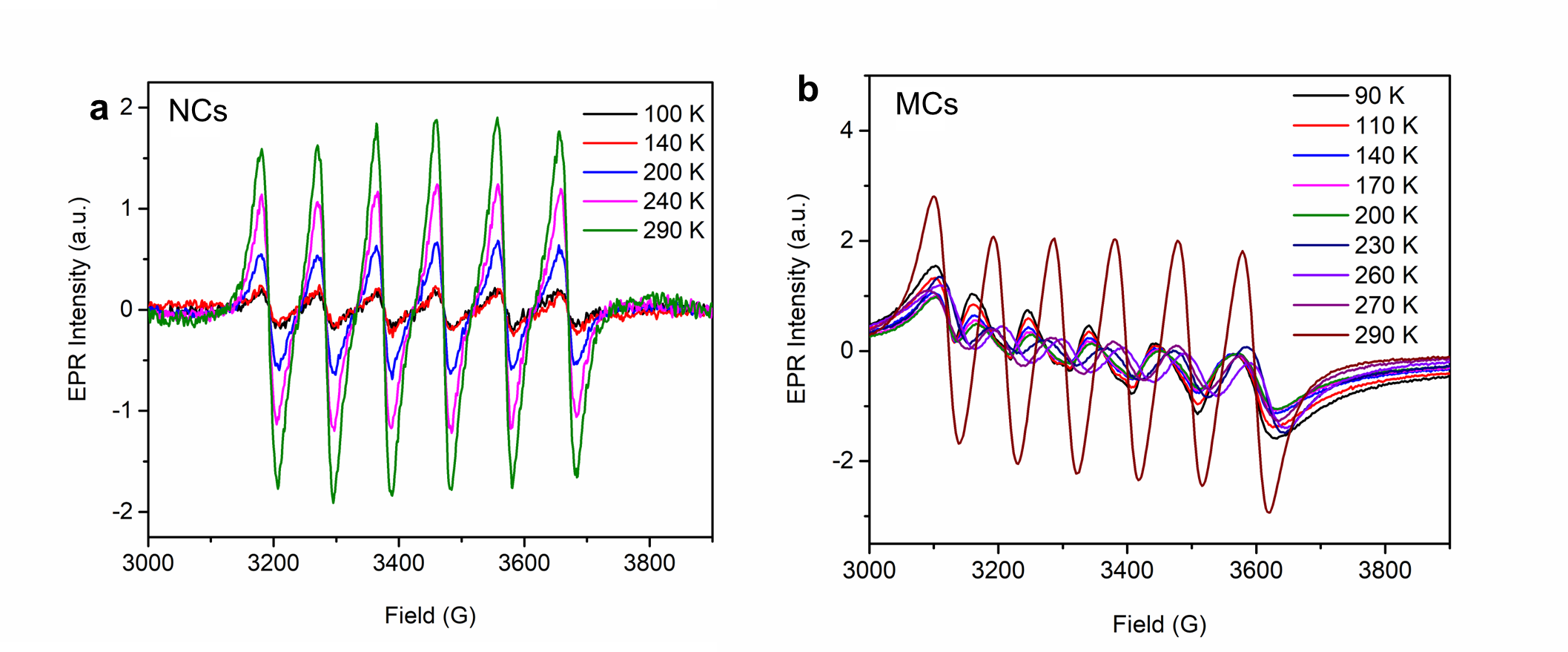


**Figure S7.** Temperature-dependent EPR spectra of perovskite NCs and self-assembled MCs.

The equation of fitting for the TA decay data is shown as equation S1:


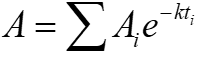
 (S1)

**Table S2.** Fitting of TA decay (Probe at 395 nm)

| Sample | A_1_ (%) | A_2_ (%) | A_3_ (%) | $\tau_{1} (ps)$ | $\tau_{2} (ps)$ | $\tau_{3} (ps)$ |
| --- | --- | --- | --- | --- | --- | --- |
| MCs | 13±0.5 | 76±1.0 | 18±0.3 | 19.47±1.25 | 2.94±0.48 | 2.16±0.32 |
| NCs | 21±1.0 | 77±1.4 | / | 21.4±0.42 | 2.79±0.17 | / |

The kinetics are fit by a multiple-exponential function: $\Delta A \left( t \right)=a_{1}\exp\left( {-t}/{\tau_{1}} \right)+a_{2}\exp\left( {-t}/{\tau_{2}} \right)+a_{3}\exp({-t}/{\tau_{3}})$, where $a_{1}$, $a_{2}$, $a_{3}$ are the amplitudes and $\tau_{1}$, $\tau_{2}$, $\tau_{3}$ are the decay time constants. These time constants are derived from the analysis of the TA decay curves (Figure 2c). The extended lifetime $\tau_{3}$ observed in the MCs is ascribed to an additional carrier recombination pathway induced by defects.

The equation of fitting of TRPL is shown as equation S2:

$t=A_{1}e^{-t/t_{1}}+A_{2}e^{-t/t_{2}}$ (S2)

**Table S3.** TRPL decay curve fitting results (Probe at 600 nm)

|  | ***NCs*** | ***MCs*** |
| --- | --- | --- |
| $\boldsymbol{A}_{\boldsymbol{1}}$ | ***0.68*** | ***0.53*** |
| $\boldsymbol{A}_{\boldsymbol{2}}$ | ***0.27*** | ***0.38*** |
| $\boldsymbol{t}_{\boldsymbol{1}}\boldsymbol{(ms)}$ | ***0.91*** | ***0.25*** |
| $\boldsymbol{t}_{\boldsymbol{2}}\boldsymbol{(ms)}$ | ***2.08*** | ***4.37*** |


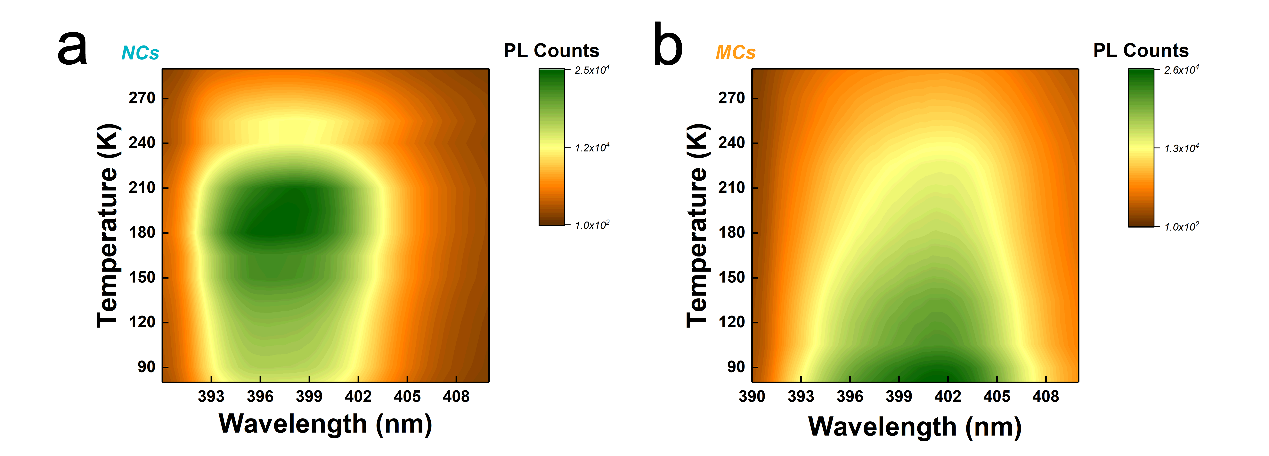


**Figure S8.** Temperature dependent PL of exciton in perovskite host of (a) NCs and (b) self-assembled MCs.





**Figure S9.** Fitting results of Huang-Rhys factor.

The equation to fit the FWHM of host lattice results by Huang-Rhys factors is shown as equation S3:


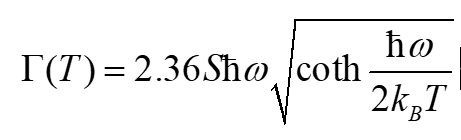
 (S3)







**Figure S10.** Temperature dependent PL of Mn^2+^ ions of different batch of perovskite MCs.

**References**

1. D. Parobek, Y. Dong, T. Qiao and D. H. Son, *Chemistry of Materials*, 2018, **30**, 2939-2944.

2. S. Das Adhikari, A. K. Guria and N. Pradhan, *The Journal of Physical Chemistry Letters*, 2019, **10**, 2250-2257.

3. S. Paul, E. Bladt, A. F. Richter, M. Doblinger, Y. Tong, H. Huang, A. Dey, S. Bals, T. Debnath, L. Polavarapu and J. Feldmann, *Angewandte Chemie International Edition*, 2020, **59**, 6794-6799.

4. G. Raino, M. A. Becker, M. I. Bodnarchuk, R. F. Mahrt, M. V. Kovalenko and T. Stoferle, *Nature*, 2018, **563**, 671-675.

5. D. Lapkin, C. Kirsch, J. Hiller, D. Andrienko, D. Assalauova, K. Braun, J. Carnis, Y. Y. Kim, M. Mandal, A. Maier, A. J. Meixner, N. Mukharamova, M. Scheele, F. Schreiber, M. Sprung, J. Wahl, S. Westendorf, I. A. Zaluzhnyy and I. A. Vartanyants, *Nature Communications*, 2022, **13**, 892.

6. C. Li, X. Li, X. Liu, L. Ma, H. Yan, L. Tong, Z. Yang, J. Liu, D. Bao, J. Yin, X. Li, P. Wang, R. Li, L. Huang, M. Yu, S. Jia, and T. Wang, ACS Nano, 2024, **18,** 9128.
